# Prognostic role of the systemic immune-inflammation index in biliary tract cancers: a meta-analysis of 3,515 patients

**DOI:** 10.1186/s12957-022-02783-z

**Published:** 2022-09-29

**Authors:** Buwen Zhang, Weiyun Yao

**Affiliations:** 1 Department of Oncology, Changxing People’s Hospital, Huzhou, 313100 Zhejiang China; 2 Department of Hepatobiliary Surgery, Changxing People’s Hospital, Huzhou, 313100 Zhejiang China

**Keywords:** Systemic immune-inflammation index, Biliary tract cancers, Meta-analysis, Prognosis, Risk factors

## Abstract

**Background:**

The systemic immune-inflammation index (SII) is an inflammatory parameter calculated as platelet count × neutrophil count/lymphocyte count in the peripheral blood. In recent years, the prognostic role of the SII in patients with biliary tract cancer (BTC) has been gradually investigated. However, the results were controversial. This meta-analysis aimed to illustrate the prognostic value of the SII in BTC.

**Methods:**

The electronic databases of PubMed, the Web of Science, Embase, and the Cochrane Library were thoroughly retrieved up to April 15, 2022. Pooled hazard ratios (HRs) with 95% confidence intervals (CIs) were used to evaluate the prognostic value of the SII for clinical outcomes. The association between the SII and overall survival (OS) and recurrence-free survival (RFS)/progression-free survival (PFS) was evaluated.

**Results:**

Thirteen studies involving 3515 patients were included in this meta-analysis. The pooled results indicated that an elevated SII was significantly associated with poor OS (HR, 1.77; 95% CI, 1.47–2.14; *p*<0.001) and RFS/PFS (HR, 1.66; 95% CI, 1.38–1.99; *p*<0.001) in patients with BTC. Subgroup analysis stratified by country, sample size, and cutoff value showed similar results. The sensitivity analysis and publication bias test confirmed the reliability of our results.

**Conclusions:**

An elevated pretreatment SII was significantly associated with worse OS and RFS/PFS in patients with BTC. Our results suggest that the SII is a valuable and cost-effective prognostic parameter for the treatment of patients with BTC.

**Supplementary Information:**

The online version contains supplementary material available at 10.1186/s12957-022-02783-z.

## Background

Biliary tract cancers (BTCs) comprise a heterogeneous group of aggressive malignancies involving the bile ducts and gallbladder [1]. BTC accounts for approximately 3% of all gastrointestinal malignancies and is the second most common primary hepatic malignancy after hepatocellular carcinoma [2]. BTC comprises intrahepatic cholangiocarcinoma (ICC), extrahepatic cholangiocarcinoma (ECC), and gallbladder cancer (GBC) [3]. The histology of BTC is mainly adenocarcinoma. The incidence of cholangiocarcinoma has been increasing worldwide, whereas that of GBC has been decreasing in recent years [4]. Surgical resection is the only method for the long-term survival of patients with resectable BTC [2]. However, approximately 80% of BTC cases are unresectable with clear margins or metastatic when diagnosed [5]. Immunotherapy for BTC has shown promising results. Durvalumab is an anti-PD-L1 inhibitor, and several clinical trials are ongoing to evaluate its efficacy in BTC [6]. The FGFR inhibitor pemigatinib was the first US Food and Drug Administration-approved molecularly targeted therapy for the treatment of cholangiocarcinoma [7]. A recent single study in Italy revealed that the timing of the first radiofrequency ablation significantly affected survival outcomes in ICC in multivariate analysis [8]. The prognosis for unresectable BTC is poor, with a 5-year survival rate of 2% [9]. Prognostic biomarkers are important for the selection of patient management strategies and prediction of clinical outcome prediction [10]. The lack of novel prognostic markers is partially responsible for the poor prognosis of patients with BTC. Therefore, identifying a cost-effective and reliable prognostic marker before treatment is important for BTC treatment.

Prolonged inflammation is a hallmark of cancer [11], and systemic immune responses participate in tumor growth and development [12]. In recent years, many inflammation-related markers have been reported as the prognostic indexes in patients with BTC, such as the neutrophil-to-lymphocyte ratio [13], platelet-to-lymphocyte ratio [14], systemic inflammation response index [15], and systemic immune-inflammation index (SII) [16–18]. The SII was first proposed in 2014 by Hu et al. to predict the prognosis of patients with hepatocellular carcinoma receiving surgical resection [19]. The SII is calculated as neutrophil × platelet/lymphocyte count. Previous studies have shown that an elevated SII is associated with poor prognosis in non-small cell lung cancer (SCLC) [20], colorectal cancer [21], breast cancer [22], and renal cell carcinoma [23]. Many studies have also investigated the prognostic significance of the SII in BTC; however, the results were inconsistent [16–18, 24–33]. Some studies identified the SII as a significant prognostic factor for BTC [27–29], whereas others reported that this association was nonsignificant [16, 33]. For example, Tsilimigras et al. reported that an elevated SII was an independent prognostic marker for overall survival (OS) in patients with ICC (hazards ratio [HR], 1.70; 95% confidence interval [CI], 1.23–2.34; *p*=0.001) [26]. Moreover, Li et al. also demonstrated that an SII of >510 was an independent predictor of OS (HR, 1.90; 95% CI, 1.42–2.54; *p*<0.001) in a multicenter study including 1072 patients with GBC [30]. However, some other studies reported that there was no significant difference between the SII and survival of patients with BTC. For example, in a recent study, Ha et al. showed that the SII was not an independent prognostic factor for OS in patients with advanced BTC in multivariate analysis (HR, 0.928; 95% CI, 0.59–1.45; *p*=0.745) [16]. Therefore, to comprehensively identify the prognostic role of the SII in patients with SII, we performed this meta-analysis.

## Methods

### Search strategy

This meta-analysis was performed in accordance with the Preferred Reporting Items for Systematic Reviews and Meta-Analyses statement [34] (Supplementary file 1). The protocol for this meta-analysis was registered in INPLASY (registration number, INPLASY202280082) and is available at https://inplasy.com/inplasy-2022-8-0082/. The electronic databases of PubMed, the Web of Science, Embase, and the Cochrane Library were thoroughly retrieved up to April 15, 2022. The following search strategies were applied: (“systemic immune-inflammation index” OR “SII” OR “systemic immune-inflammatory index”) AND (“biliary tract cancer” OR “bile duct cancer” OR “bile duct neoplasms” OR “cholangiocarcinoma” OR “gallbladder cancer” OR “gallbladder carcinoma”). All searches were performed using a combination of MeSH terms and free-test words. Only studies published in English were considered. The references of the retrieved studies were manually examined to identify other potential inclusions.

### Inclusion and exclusion criteria

The inclusion criteria were as follows: (i) studies reported the relationship between the SII and survival outcomes of patients with BTC, including OS, progression-free survival (PFS), recurrence-free survival (RFS), and disease-free survival (DFS); (ii) the diagnosis of BTC was pathologically confirmed; (iii) a definite cutoff value of the SII was provided; (iv) the HRs with 95% CIs of prognostic factors could be extracted, or sufficient data were provided to calculate them; and (v) studies were published in English. The exclusion criteria were as follows: (i) nonhuman studies; (ii) reviews, letters, comments, case reports, and meeting abstracts; (iii) studies with overlapping patients; and (iv) studies without sufficient data.

### Data extraction and quality assessment

Two independent investigators (BZ and WY) extracted the necessary data from the eligible studies, and all disagreements were resolved through discussion to reach a consensus. The following data were extracted: name of the first author, year of publication, country, study design, sample size, histological type, tumor stage, treatment, follow-up, cutoff value of the SII, survival endpoint, survival analysis type, and HR and 95% CI. The quality of the included studies was evaluated using the Newcastle–Ottawa Scale (NOS) [35]. The NOS scores ranged from 0 to 9, and studies with NOS scores of ≥6 were regarded as high-quality studies.

### Statistical analysis

Pooled HRs and 95% CIs were used to evaluate the prognostic value of the SII for clinical outcomes. Heterogeneity across studies was evaluated using the chi-square *Q* test and *I*^2^ index. If low heterogeneity between studies (Ph>0.10, *I*^2^ < 50%) was observed, a fixed-effects model was applied for analysis. Otherwise, a random-effects model was used. Subgroup analysis stratified by various clinicopathological factors was performed to identify the source of heterogeneity. Sensitivity analysis was performed by sequentially omitting each study to observe the impact of individual studies on the overall results. Funnel plots and Begg’s and Egger’s tests were used to examine potential publication bias. Stata software (version 12.0; Stata Corporation, College Station, TX, USA) was used for all statistical analyses. Statistical significance was set at *p*<0.05.

### Ethics statement

Ethical approval was not required for this study because the data from this meta-analysis were based on previous studies and no individual patient information was used.

## Results

### Literature selection

**Fig. 1:**
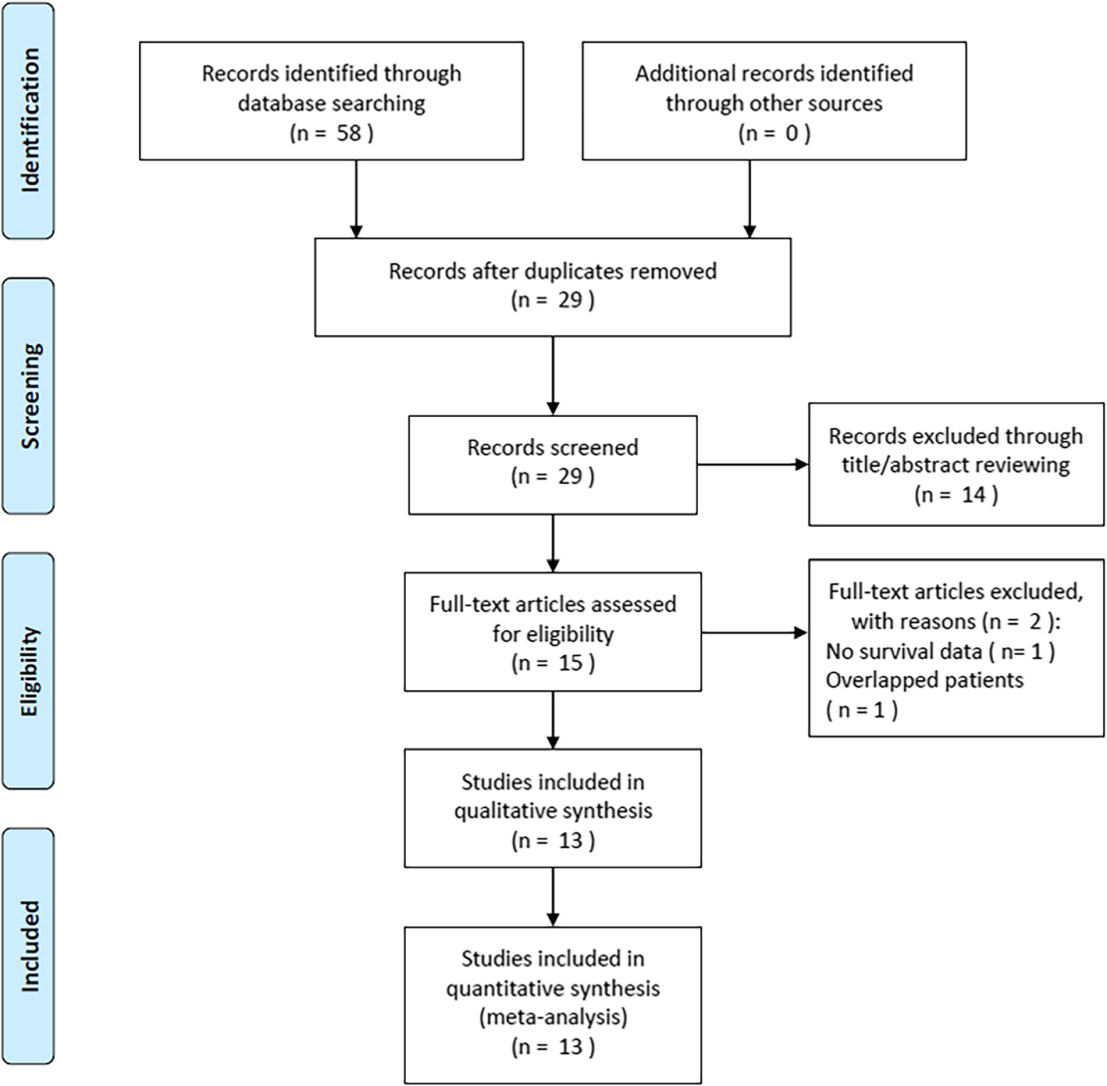
Flow diagram of the literature search and selection

An initial literature search identified 58 records (Fig. 1). After removing duplicate studies, 29 studies remained. Fourteen studies were excluded after reviewing the titles and abstracts, and 15 studies were further evaluated by full-text examination. Subsequently, two studies were eliminated because one study did not provide survival data and the other included overlapping patients. Finally, 13 studies with 3515 patients [16–18, 24–33] were included in this meta-analysis (Fig. 1).

### Characteristics of included studies

The basic characteristics of the 13 included studies [16–18, 24–33] are shown in Table 1. They were published between 2016 and 2022 and were retrospective studies. Eight studies were performed in China [17, 24, 25, 27–31], two in the USA [18, 26], and one each in Korea [16], Japan [32], and Italy [33]. The total sample size was 3515, ranging from 28 to 1072. The median sample size was 140 patients. Five studies included patients with ICC [18, 24, 26, 27, 31], three studies enrolled patients with ECC [17, 32, 33], three studies recruited patients with GBC [25, 28, 30], and two studies included patients with BTC [16, 29]. All included studies reported the prognostic value of the SII for OS in BTC, five studies reported the association between the SII and RFS [24, 26, 27, 31, 33], and one study showed a correlation between the SII and PFS [29]. The cutoff values of the SII ranged from 447.48 to 1450, and the median value was 600. Nine studies reported the HRs and 95% CIs from a multivariate analysis [16, 24–28, 30–32], and four studies presented the HRs and 95% CIs from univariate analysis [17, 18, 29, 33]. The NOS scores of the included studies ranged from 6 to 8, with a median value of 7, indicating that all included studies were of high quality (Table 1).

**Table 1:** Baseline characteristics of included studies in this meta-analysis

Study	Year	Country	Study period	Study design	Sample size	Age (years) Median (range)	Sex (M/F)	Histology	TNM stage	Treatment	Follow-up (months) Median (range)	Cut-off value of SII	Survival endpoints	Survival analysis	NOS score
Ha, H.	2016	Korea	2004–2009	Retrospective	158	59.6 (31.3–76.2)	103/55	BTC	IV	Chemotherapy	95.3	572.38	OS	Multivariate	7
Hu, X.	2019	China	2012–2017	Retrospective	113	70	75/38	ECC	I–IV	PTBS + ^125^I	To Sep 2017	456	OS	Univariate	7
Sellers, C. M.	2019	USA	2005–2016	Retrospective	131	65 (57–71)	68/63	ICC	I–IV	Surgery	12	867	OS	Univariate	8
Li, H.	2020	China	2009–2017	Retrospective	530	57.2	256/274	ICC	I–III	Surgery	18.0 (1.0-115.4)	450	OS, RFS	Multivariate	7
Sun, L.	2020	China	2003–2017	Retrospective	142	63	60/82	GBC	I–IV	Surgery	12	600	OS	Multivariate	8
Tsilimigras, D. I.	2020	USA	2000–2017	Retrospective	688	57 (49–65)	416/272	ICC	I–III	Surgery	22.3	1,150	OS, RFS	Multivariate	8
Zhang, Z.	2020	China	2013–2017	Retrospective	128	56.19	70/58	ICC	I–III	Surgery	25.2	1,027	OS, RFS	Multivariate	7
Chen, H.	2021	China	2012–2020	Retrospective	93	62 (32–90)	62/31	GBC	I–III	Surgery	14 (2-60)	824	OS	Multivariate	8
Du, F.	2021	China	2016–2019	Retrospective	60	61 (28–83)	41/19	BTC	IV	Immunotherapy	To Apr 2020	710	OS, PFS	Univariate	6
Li, L.	2021	China	2002–2019	Retrospective	1072	62	414/658	GBC	I–III	Surgery	53.8	510	OS	Multivariate	7
Ren, A.	2021	China	2013–2018	Retrospective	28	51.5 (46.8–60)	25/3	ICC	IV	Liver transplantation	33.5	447.48	OS, RFS	Multivariate	8
Terasaki, F.	2021	Japan	2002–2015	Retrospective	140	71 (39–85)	109/31	ECC	I–III	Surgery	48.2	1,450	OS	Multivariate	8
Di Martino, M.	2022	Italy	2010–2019	Retrospective	232	70	146/86	ECC	I–IV	Surgery	35.8	592	OS, RFS	Univariate	7

*M* Male, *F* Female, *BTC* Biliary tract cancer, *ECC* Extrahepatic cholangiocarcinoma, *ICC* Intrahepatic cholangiocarcinoma, *GBC* Gallbladder cancer, *PTBS* Percutaneous transhepatic biliary stenting, *OS* Overall survival, *PFS* Progression-free survival, *RFS* Recurrence-free survival, *NOS* Newcastle-Ottawa Scale

### SII and OS in BTC

**Fig. 2:**
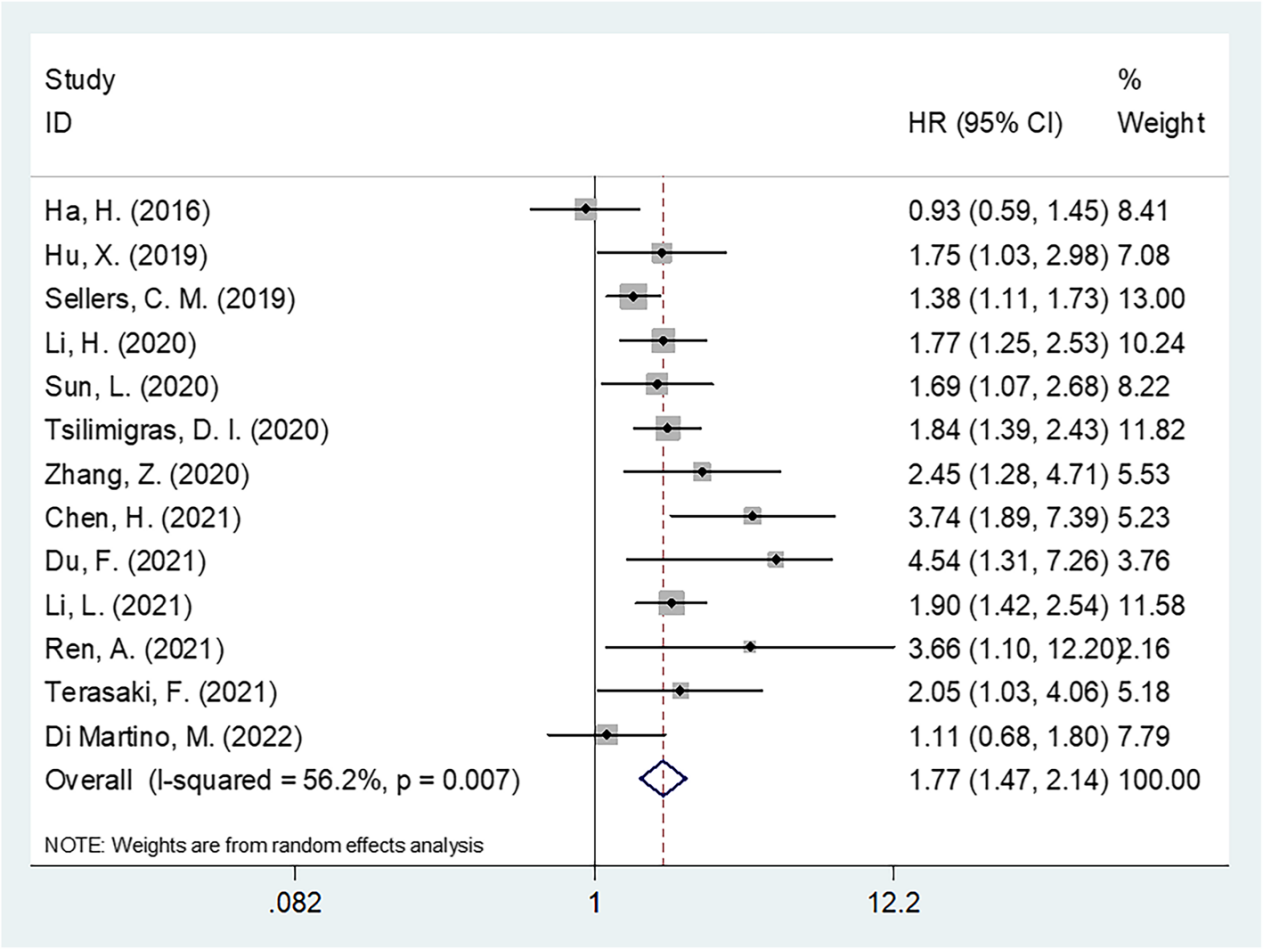
Forest plot for the relationship SII and OS in BTC patients

All 13 studies with 3515 patients [16–18, 24–33] showed a connection between the SII and OS in patients with BTC. Because of the significant heterogeneity (*I*^2^=56.2% and *p* for heterogeneity=0.007), a random-effects model was applied. As shown in Table 2 and Fig. 2, the combined results were as follows: HR, 1.77, and 95% CI, 1.47–2.14 (*p*<0.001), indicating that a high SII was significantly associated with poor OS in BTC. Subgroup analysis demonstrated that the prognostic value of the SII for OS was still significant irrespective of country, sample size, cutoff value, or survival analysis type (Table 2).

**Table 2:** Subgroup analysis of the prognostic effect of SII for OS in patients with BTC

Subgroups	No. of studies	No. of patients	HR (95%CI)	*p*	Effects model	Heterogeneity	
						*I*^2^(%)	Ph
Total	13	3515	1.77 (1.47–2.14)	<0.001	Random	56.2	0.007
Countries							
Asian	10	2,464	1.97 (1.54–2.54)	<0.001	Random	54.3	0.020
Non-Asian	3	1,051	1.47 (1.14–1.90)	0.003	Random	50.9	0.130
Sample size							
≤140	7	693	2.31 (1.59–3.36)	<0.001	Random	63.7	0.011
>140	6	2,822	1.55 (1.25–1.93)	<0.001	Random	52.4	0.062
Histology							
BTC	2	218	1.97 (0.42–9.30)	0.394	Random	90.3	0.001
ECC	3	485	1.49 (1.08–2.05)	0.014	Fixed	23.9	0.269
ICC	5	1,505	1.65 (1.42–1.92)	<0.001	Fixed	36.5	0.178
GBC	3	1,307	2.00 (1.58–2.51)	<0.001	Fixed	48.2	0.145
TNM stage							
I–III	6	2,651	1.96 (1.67–2.30)	<0.001	Fixed	0	0.483
I–IV	4	618	1.42 (1.19–1.69)	<0.001	Fixed	0	0.524
IV	3	246	2.33 (0.72–7.57)	0.160	Random	84.5	0.002
Treatment							
Surgery	10	3,184	1.70 (1.51–1.92)	<0.001	Fixed	41.8	0.079
Non-surgery	3	331	1.81 (0.82–4.04)	0.144	Random	82.0	0.004
Cutoff value							
≤600	7	2,275	1.59 (1.35–1.87)	<0.001	Fixed	47.8	0.074
>600	6	1,240	2.17 (1.55–3.03)	<0.001	Random	67.1	0.009
Survival analysis							
Multivariate	9	2,979	1.81 (1.57–2.09)	<0.001	Fixed	47.8	0.053
Univariate	4	536	1.64 (1.10–2.45)	0.016	Random	65.5	0.034

*BTC* Biliary tract cancer, *ECC* Extrahepatic cholangiocarcinoma, *ICC* Intrahepatic cholangiocarcinoma, *GBC* Gallbladder cancer, *OS* Overall survival

### SII and RFS/PFS in BTC

**Fig. 3:**
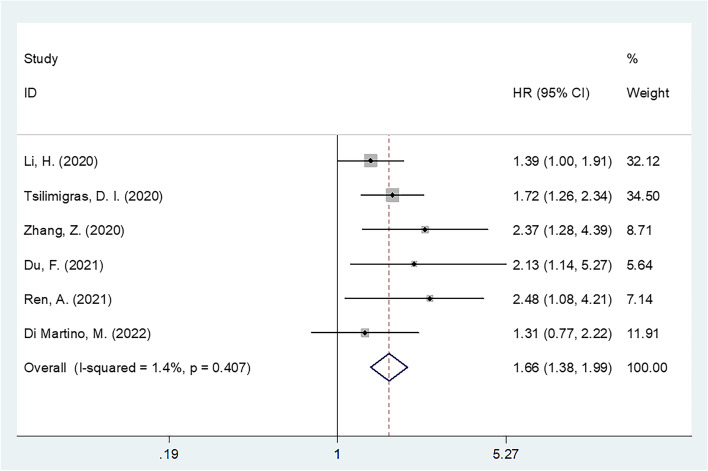
Forest plot for the relationship SII and RFS/PFS in BTC patients

Six studies with 1666 patients [24, 26, 27, 29, 31, 33] showed an association between the SII and RFS/PFS in BTC. Heterogeneity was not significant (*I*^2^=1.4% and *p* for heterogeneity=0.407), and a fixed-effects model was applied. The combined results were as follows: HR, 1.66, and 95% CI, 1.38–1.99 (*p*<0.001) (Table 3, Fig. 3), which suggested that an elevated SII was associated with poor RFS/PFS in BTC. Subgroup analysis demonstrated that the prognostic role of the SII for RFS/PFS was not affected by country, sample size, or cutoff value (Table 3).

**Table 3:** Subgroup analysis of the prognostic effect of SII for RFS/PFS in patients with BTC

Subgroups	No. of studies	No. of patients	HR (95%CI)	*p*	Effects model	Heterogeneity	
						*I*^2^(%)	Ph
Total	6	1666	1.66 (1.38–1.99)	<0.001	Fixed	1.4	0.407
Countries							
Asian	4	746	1.71 (1.33–2.19)	<0.001	Fixed	28.6	0.241
Non-Asian	2	920	1.60 (1.23–2.09)	0.001	Fixed	0	0.384
Sample size							
≤140	3	216	2.34 (1.58–3.46)	<0.001	Fixed	0	0.955
>140	3	1450	1.51 (1.23–1.85)	<0.001	Fixed	0	0.539
Histology							
BTC	1	60	2.13 (0.99–4.57)	0.054	-	-	-
ECC	1	232	1.31 (0.77–2.22)	0.314	-	-	-
ICC	4	1374	1.69 (1.38–2.06)	<0.001	Fixed	22.6	0.275
TNM stage							
I–III	3	1346	1.63 (1.32–2.01)	<0.001	Fixed	20.5	0.284
I–IV	1	232	1.31 (0.77–2.22)	0.314	Fixed	-	-
IV	2	88	2.32 (1.39–3.86)	0.001	Fixed	0	0.765
Treatment							
Surgery	5	1606	1.64 (1.36–1.97)	<0.001	Fixed	13.9	0.325
Non-surgery	1	60	2.13 (0.99–4.57)	0.054	-	-	-
Cut-off value							
≤600	3	790	1.48 (1.15–1.91)	0.002	Fixed	22.9	0.273
>600	3	876	1.87 (1.44–2.42)	<0.001	Fixed	0	0.622
Survival analysis							
Multivariate	4	1374	1.69 (1.38–2.06)	<0.001	Fixed	22.6	0.275
Univariate	2	292	1.53 (0.99–2.36)	0.054	Fixed	3.6	0.308

*BTC* Biliary tract cancer, *ECC* Extrahepatic cholangiocarcinoma, *ICC* Intrahepatic cholangiocarcinoma, *PFS* Progression-free survival, *RFS* Recurrence-free survival

### Sensitivity analysis

**Fig. 4:**
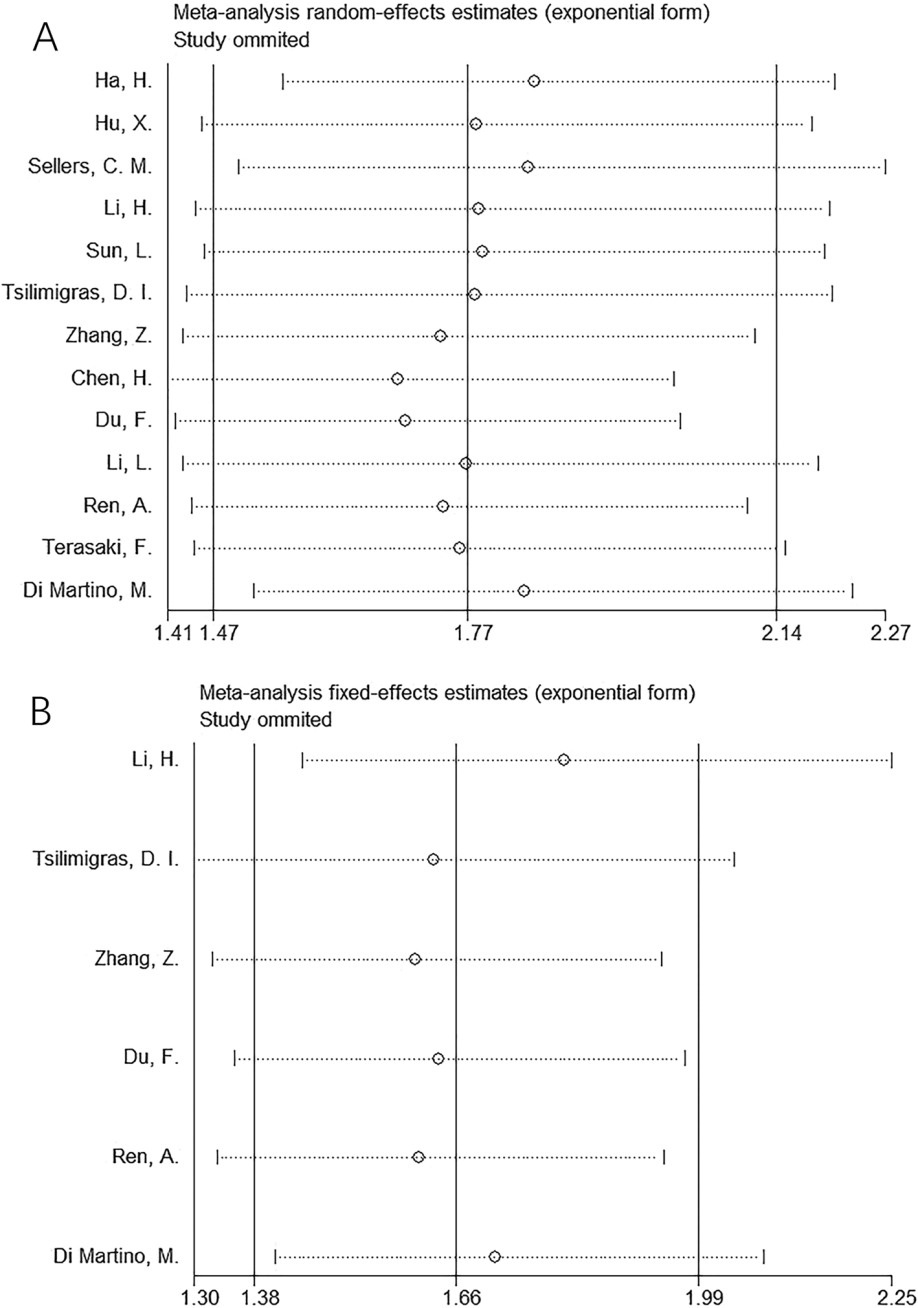
Result of sensitivity analyses by omitting one study in each turn for (**A**) OS and (**B**) RFS/PFS

Sensitivity analysis was performed to explore potential sources of heterogeneity for OS and RFS/PFS. As shown in Fig. 4, the pooled HRs and corresponding 95% CIs were stable in our meta-analysis.

### Publication bias

**Fig. 5:**
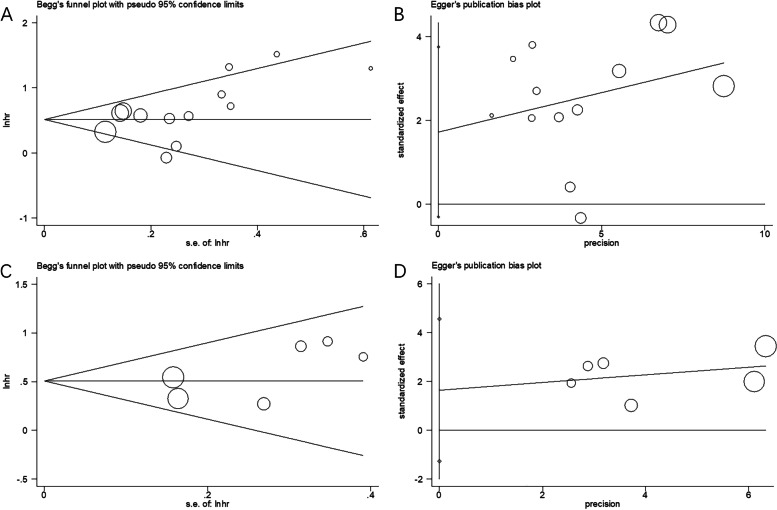
Publication bias test by Begg’s funnel plot and Egger’s test. **A** Begg’ test for OS, *p*=0.079; **B** Egger’s test for OS, *p*= 0.088; **C** Begg’ test for RFS/PFS, *p*=0.260; **D** Egger’s test for RFS/PFS, *p*= 0.193

Begg’s and Egger’s tests were conducted to examine the potential publication bias in this meta-analysis. As shown in Fig. 5, the results of Begg’s and Egger’s tests showed that there was no significant publication bias for OS (Begg’s test, *p*=0.079; Egger’s test, *p*=0.088) or RFS/PFS (Begg’s test, *p*=0.260; Egger’s test, *p*=0.193).

## Discussion

To our knowledge, this is the first meta-analysis to assess the prognostic value of the SII in stratifying the prognosis of patients with BTC. Many studies have explored the prognostic significance of the SII in patients with BTC [16–18, 24–33]; however, the results have been inconsistent. In the current meta-analysis, we included 13 studies with 3515 patients to shed light on this issue. The results suggested that an elevated SII was significantly associated with worse OS and RFS/PFS in patients with BTC. Moreover, the prognostic value of the SII was not influenced by country, sample size, cutoff value, or survival analysis type in the subgroup analysis. The sensitivity analysis and publication bias test showed that our results were reliable. Taken together, this meta-analysis proposes that the SII could be a promising prognostic biomarker for survival prediction in patients with BTC.

Systemic inflammatory responses can promote tumor invasion and progression by reducing apoptosis and promoting metastasis [36]. The SII is a parameter reflecting inflammatory status and is composed of three inflammatory immune cell counts: neutrophil, lymphocyte, and platelet. The SII was defined as platelet count × neutrophil count/lymphocyte count [37]; therefore, an increase in platelet and neutrophil counts and/or a decrease in lymphocyte counts can lead to a high SII. Neutrophils can promote proliferation and metastasis of BTC through multiple mechanisms [38]. Neutrophils can inhibit the host immune response to cancer cells by suppressing cytotoxic immune cells via the secretion of various cytokines and chemokines [39]. Elevated platelet levels have been shown to accelerate tumor angiogenesis and prevent cytolysis [40]. Platelets can also inhibit tumor cell extravasation by potentiating tumor cell-induced endothelial cell retraction and, therefore, contribute to the promotion of tumor cell proliferation and metastasis [41]. In contrast, lymphocytes play an important role in T cell-mediated antitumor responses. Lymphocytes can change the tumor microenvironment and prevent tumorigenesis and tumor relapse by migrating and infiltrating the tumor microenvironment [42]. Therefore, the SII combines the significance of neutrophil, platelet, and lymphocyte counts and is a promising prognostic biomarker.

In addition to cancer, the SII has also been reported as a significant prognostic marker in other diseases. For example, a recent retrospective cohort study showed that the SII was associated in a J-shaped pattern with all-cause mortality among critically ill patients with acute kidney injury [43]. Another study indicated that patients with heart failure with higher SII values had a shorter survival time [44]. Xia et al. demonstrated that the SII is a potential new diagnostic biomarker in patients with severe COVID-19 in a study including 125 patients diagnosed with COVID-19 [45].

Previous meta-analyses have also investigated the prognostic impact of the SII in a variety of cancer types [46–48]. Li et al. showed that an elevated preoperative SII was significantly associated with worse survival outcomes and adverse pathological features in patients with bladder cancer based on a meta-analysis of 7087 patients [49]. Fu et al. reported that a higher SII value was significantly associated with worse OS and DFS in gastric cancer in a meta-analysis of 11 studies [50]. Another meta-analysis of 3180 patients revealed that a high SII was independently associated with poor survival outcomes in patients with renal cell carcinoma [51]. A recent meta-analysis indicated that a high SII was significantly associated with OS in patients with SCLC [52]. Moreover, a high SII was correlated with extensive-stage SCLC [52]. The results of our meta-analysis are in line with those of the prognostic role of the SII in other solid tumors [49, 51, 52].

This meta-analysis has several limitations. First, all included studies had a retrospective design. Therefore, there may have been a potential selection bias. Second, the population included in this meta-analysis was mainly from Asian countries but is not a good representation of the worldwide population. Third, the cutoff values of the SII varied across the included studies, which may have introduced heterogeneity in the meta-analysis. Therefore, large-scale prospective trails using uniform SII cutoff value are needed to consolidate our findings.

Notably, inherent heterogeneity may have existed in our meta-analysis, and we performed several analyses to reveal the impact of heterogeneity on our results. First, in the data analysis (Tables 2 and 3), we selected a fixed-effects or random-effects model according to the level of heterogeneity. Second, the sensitivity analysis (Fig. 4) showed that the overall results were not influenced by a single study. Third, the publication bias test demonstrated that there was no significant publication bias in our meta-analysis (Fig. 5). Therefore, the aforementioned analysis suggests that there was inherent heterogeneity in our meta-analysis; however, our results were reliable and were not affected by this heterogeneity.

## Conclusions

In summary, an elevated pretreatment SII was significantly associated with worse OS and RFS/PFS in patients with BTC. Our results suggest that the SII is a valuable and cost-effective prognostic parameter for the treatment of patients with BTC.

## Supplementary Information

**Additional file 1.** The PRISMA checklist.

## Data Availability

The information used and analyzed during this study is available from the original literature listed in the reference. The datasets analyzed during the current study are available from the corresponding author on reasonable request.

## Abbreviations

SII: Systemic immune-inflammation index
BTC: Biliary tract cancers
HR: Hazards ratio
CI: Confidence interval
OS: Overall survival
PFS: Progression-free survival
RFS: Recurrence-free survival
DFS: Disease-free survival
NOS: Newcastle-Ottawa Scale
